# Insulin Resistance Indices Predict Mortality in Cardiovascular Disease: A Large‐Scale NHANES Study With Machine Learning Validation

**DOI:** 10.1002/fsn3.71080

**Published:** 2025-11-19

**Authors:** Zeyi Zhou, QiuJu Ding, Xinlong Tang, Lixiang Han, Yali Wang, Jintao Qian, Kai Li, Qing Zhou

**Affiliations:** ^1^ Department of Cardiovascular Surgery, Nanjing Drum Tower Hospital, Affiliated Hospital of Medical School Nanjing University Nanjing China

**Keywords:** cardiovascular disease, insulin resistance, machine learning, McAuley index, METS‐IR, NHANES, SHAP analysis

## Abstract

Insulin resistance (IR) is a key driver of cardiovascular disease (CVD), the leading cause of global mortality. This study evaluated the prognostic value of two surrogate IR indices—the McAuley index and the Metabolic Score for Insulin Resistance (METS‐IR)—for predicting all‐cause and CVD mortality. Data from 22,308 NHANES participants with established CVD (1999–2018) was analyzed. Outcomes were all‐cause and CVD mortality. Cox proportional hazards models and restricted cubic splines assessed associations, while machine learning methods (random forest, XGBoost, CoxBoost, DeepHit) evaluated predictive performance. Model interpretability was assessed using SHapley Additive exPlanations (SHAP). Over a median 9.2‐year follow‐up, 3484 deaths occurred, including 1093 from CVD. A higher McAuley Index was inversely associated with risk, with each 1‐unit increase predicting a 9.2% reduction in all‐cause and 11.3% reduction in CVD mortality. Higher METS‐IR values were associated with increased mortality. Restricted cubic spline analysis confirmed significant U‐shaped relationships. Across nine models, the Cox model demonstrated the best performance (C‐index: 0.87 for all‐cause and 0.85 for CVD mortality), with time‐dependent AUCs consistently above 0.8. SHAP analysis highlighted the McAuley Index and METS‐IR as leading predictors. The McAuley Index and METS‐IR are robust, independent predictors of all‐cause and CVD mortality. Their integration with interpretable machine learning enhances risk stratification, underscoring the role of metabolic dysfunction and central adiposity in long‐term outcomes. These indices may help identify high‐risk patients who could benefit from targeted interventions.

## Graphical abstract

This study analyzed data from 22,308 participants in NHANES (1999–2018) with a median follow‐up of 9.2 years. Two insulin resistance indices—METS‐IR and McAuley index—were evaluated using nine machine learning models. METS‐IR showed a negative association with all‐cause and cardiovascular mortality and achieved the best predictive performance (C‐index: 0.87 for all‐cause, 0.85 for CVD mortality; time‐dependent AUCs > 0.8 at 1, 3, 5, and 10 years). In contrast, the McAuley index demonstrated a positive association with mortality outcomes.

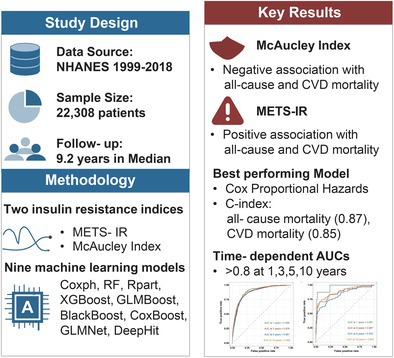

## 1 Introduction

Insulin resistance (IR) represents a critical pathophysiological mechanism underlying numerous chronic diseases, prominently featuring as both a risk factor and potentiator for cardiovascular disease (CVD) (Abdul‐Ghani et al. 2024; Sattar et al. 2020). This metabolic dysregulation, characterized by diminished cellular response to insulin, affects approximately 25%–30% of the global adult population, with prevalence increasing steadily across both developed and developing nations (Romeo et al. 2025; Younossi et al. 2019). The clinical significance of insulin resistance extends beyond its direct metabolic consequences, as it independently contributes to chronic low‐grade inflammation, oxidative stress, mitochondrial dysfunction, and dysregulation of multiple signaling pathways across various organ systems (Steinberg et al. 2025; Verkerke et al. 2024). Among the IR‐related diseases, CVD remains the leading cause of mortality worldwide, accounting for approximately 17.9 million deaths annually—representing 32% of all global deaths (Huang et al. 2022; Wieckowska‐Gacek et al. 2021). This substantial public health burden necessitates the development and validation of accessible insulin resistance indices to accurately identify high‐risk individuals and guide personalized preventive strategies that could significantly reduce CVD mortality and improve overall survival outcomes.

The quantification of insulin resistance presents significant clinical challenges, as the gold standard measurement—the hyperinsulinemic–euglycemic clamp technique—is invasive, time‐consuming, and impractical for routine clinical use (Donga et al. 2015). Consequently, several surrogate indices have been developed to estimate insulin resistance using more accessible clinical and laboratory parameters. Among these, the McAuley index, derived from fasting insulin and triglyceride levels, and the Metabolic Score for Insulin Resistance (METS‐IR), calculated from triglycerides, glucose, body mass index (BMI), and high‐density lipoprotein cholesterol (HDL‐C), have emerged as promising alternatives (Antonio‐Villa et al. 2020; Bello‐Chavolla et al. 2019; Pilz et al. 2014). The McAuley index has demonstrated a strong correlation with clamp‐derived measurements in various populations (Sarafidis et al. 2007), while METS‐IR offers the advantage of not requiring insulin measurement, making it particularly suitable for large‐scale epidemiological studies and resource‐limited settings (Cheng et al. 2023; He et al. 2024). Despite these advantages, the prognostic value of these indices in predicting all‐cause and CVD mortality remains incompletely characterized. Elucidating the relationship between these accessible insulin resistance metrics and mortality outcomes could substantially enhance risk assessment and guide preventive strategies in clinical practice.

Modern analytical approaches, including machine learning (ML) algorithms and interpretable artificial intelligence methods, offer unprecedented capabilities to model complex biological relationships and extract meaningful insights from large, multidimensional datasets (Camacho et al. 2018; Kingsmore et al. 2021). Machine learning approaches offer enhanced capabilities for modeling physiological systems by effectively capturing complex non‐linear relationships and interactions between multiple variables, which are particularly valuable when analyzing multifaceted conditions like cardiovascular disease with numerous interrelated risk factors. Machine learning algorithms, including random forests, gradient boosting machines, and deep learning architectures, can effectively identify intricate relationships between risk factors and outcomes without requiring a priori specification of model form. Furthermore, SHapley Additive exPlanations (SHAP) analysis provides a framework for interpreting these complex models by quantifying the contribution of each feature to the prediction for individual instances (Liao et al. 2024), thereby bridging the gap between predictive performance and clinical interpretability (Portlock et al. 2025). In this study, we examined the associations between two insulin resistance indices—the McAuley index and METS‐IR—and mortality outcomes, including all‐cause and cardiovascular disease (CVD) mortality, using data from the National Health and Nutrition Examination Survey (NHANES). We evaluated the predictive performance of several machine learning algorithms for mortality prediction. Furthermore, we explored the relative importance and non‐linear relationships of these indices with mortality outcomes through SHAP analysis and restricted cubic spline modeling.

## 2 Materials and Methods

### 2.1 Study Population

We used data from the National Health and Nutrition Examination Survey (NHANES), a nationally representative cross‐sectional survey designed to assess the health and nutritional status of adults and children in the United States (Force et al. 2022; Victora et al. 2022). NHANES collects data through structured interviews, physical examinations, and laboratory assessments, and provides mortality follow‐up through linkage with the National Death Index. This study was conducted in accordance with the Strengthening the Reporting of Observational Studies in Epidemiology (STROBE) guidelines.

**FIGURE 1:**
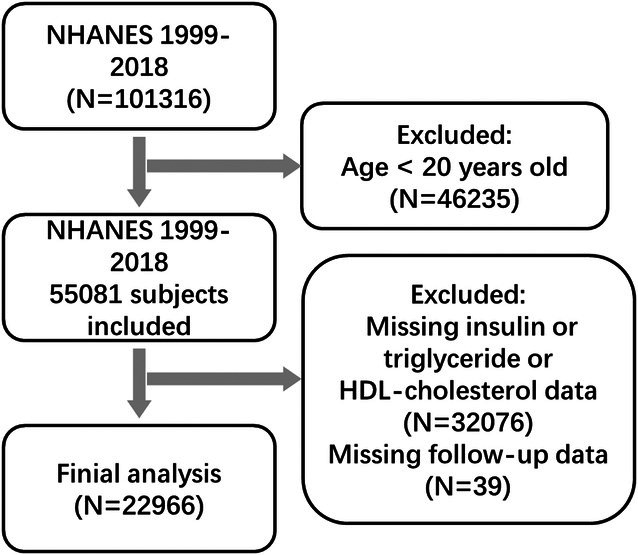
Flowchart illustrating the screening and selection process of study participants.

For this analysis, we included adult participants (aged ≥ 20 years) from 10 NHANES survey cycles (1999–2018) who had established cardiovascular disease (CVD), defined as a self‐reported history of coronary heart disease, myocardial infarction, stroke, or heart failure, as documented on the NHANES website (https://www.cdc.gov/nchs/nhanes/index.htm). Participants were excluded if they had missing data on any of the following variables: (1) fasting insulin; (2) triglycerides (TG); (3) high‐density lipoprotein cholesterol (HDL‐C); (4) body mass index (BMI); or (5) follow‐up status. After applying these criteria, a total of 22,308 eligible participants were included. The selection process is outlined in Figure 1.

### 2.2 Calculation of Insulin Resistance Indices

The McAuley index was calculated using the formula:

$$\text{McAuley}=e^{\left(2.63-0.28\ln I-0.31\ln T\right)}$$

where *I* is fasting insulin concentration (mIU/L), and *T* is fasting triglyceride concentration (mmol/L). Lower values of the McAuley index indicate higher insulin resistance.

METS‐IR was calculated as:

$$\text{METS}-\mathrm{IR}=\frac{\left[\ln\left(2\mathrm{FG}+\mathrm{TG}\right)\right]\times \mathrm{BMI}}{\ln\left(\mathrm{HDL}-C\right)}$$

where FG is fasting glucose (mg/dL), TG is triglycerides (mg/dL), HDL‐C is high‐density lipoprotein cholesterol (mg/dL), and BMI is body mass index (kg/m^2^). Higher METS‐IR values represent greater insulin resistance.

### 2.3 Outcomes

The primary outcomes were all‐cause and cardiovascular mortality. Mortality status and causes of death were determined through linkage with the National Death Index, with follow‐up extended through the date of death or until December 31, 2019. All‐cause mortality was defined as death from any cause, including heart disease, malignant neoplasms, chronic lower respiratory diseases, unintentional injuries, cerebrovascular diseases, Alzheimer's disease, diabetes mellitus (DM), influenza or pneumonia, kidney disease, or other causes. Cardiovascular mortality was defined as death due to heart disease or cerebrovascular disease, coded as *UCOD_LEADING = 001* (heart disease) or *005* (cerebrovascular disease).

### 2.4 Covariates

Covariates included sociodemographic, clinical, laboratory, and medical history variables. Sociodemographic variables comprised age, sex, race/ethnicity, education level, and marital status. Clinical variables included BMI, waist circumference, waist‐to‐height ratio (WHtR), smoking status, alcohol consumption, and physical activity. Laboratory measurements included low‐density lipoprotein (LDL) cholesterol, high‐density lipoprotein (HDL) cholesterol, triglycerides, fasting glucose, fasting insulin, hemoglobin, hemoglobin A1c (HbA1c), C‐reactive protein (CRP), albumin, and white blood cell (WBC) count. Medical history variables included hypertension, diabetes mellitus, hyperlipidemia, and atherosclerotic cardiovascular disease (ASCVD).

### 2.5 Statistical Analysis

#### 2.5.1 Descriptive Statistics

Baseline characteristics were summarized by quartiles of the McAuley index and METS‐IR. Continuous variables were presented as means ± standard deviations or medians with interquartile ranges, depending on their distribution. Categorical variables were expressed as frequencies and percentages. Differences across quartiles were assessed using analysis of variance (ANOVA) or the Kruskal–Wallis test for continuous variables, and the chi‐square test for categorical variables.

#### 2.5.2 Survival Analysis

Cox proportional hazards regression models were used to estimate hazard ratios (HRs) and 95% confidence intervals (CIs) for the associations of the McAuley index and METS‐IR with all‐cause and cardiovascular mortality, adjusting for covariates across multiple models. Kaplan–Meier survival curves were constructed to illustrate survival differences across quartiles of each index, with statistical significance assessed by log‐rank tests.

Stratified and interaction analyses were performed to examine potential effect modification by subgroups including age, sex, diabetes, hypertension, hyperlipidemia, and ASCVD. To evaluate and visualize potential nonlinear dose–response relationships between the indices and mortality outcomes, restricted cubic spline (RCS) models were employed, with knots placed at the 10th, 50th, and 90th percentiles. A two‐sided *p* < 0.05 was considered statistically significant.

### 2.6 Machine Learning Models

In our analysis, we employed a suite of machine learning models for survival analysis using the mlr3 package in R. The following algorithms were implemented and evaluated:

- Cox Proportional Hazards (Coxph): a widely used statistical method for modeling the time until an event occurs based on predictor variables.
- Random Forest (ranger): an ensemble learning method that constructs multiple decision trees and outputs the mode of the classes or mean prediction of the individual trees.
- Gradient Boosting (xgboost.cox): a powerful ensemble learning technique that builds models iteratively, with each new model correcting the errors made by the previous ones, specifically adapted for Cox regression in this case.
- Boosted Generalized Linear Model (glmboost): an approach that combines the principles of generalized linear models with boosting techniques to improve prediction accuracy.
- Regularized Generalized Linear Model (glmnet): a linear model that incorporates regularization (Lasso and/or Ridge penalties) to prevent overfitting and perform feature selection.
- Likelihood‐based Boosting (coxboost): a boosting algorithm specifically designed for Cox regression, directly optimizing the partial likelihood.
- Deep Neural Network for Survival Analysis (deephit): a neural network‐based model designed to directly estimate the hazard function and survival probabilities.
- Survival Tree (rpart): a decision tree algorithm adapted for survival data, capable of handling censored observations.
- Blackboost: another boosting algorithm based on generalized additive models, suitable for survival analysis.

Model performance was evaluated using repeated five‐fold cross‐validation. Specifically, a five‐fold cross‐validation was performed with five independent repetitions (5 × 5 CV) to provide a more robust and stable estimate of model generalizability. The mean area under the receiver operating characteristic curve (AUC) and the concordance index (C‐index) were used to assess predictive performance. The model with the highest C‐index was selected as the best performing algorithm. For each model, various combinations of hyperparameters were evaluated to identify the optimal configuration. The detailed hyperparameter settings are provided in Table S1. The hyperparameter configuration achieving the highest C‐index was adopted for cross‐model comparisons.

### 2.7 SHAP Analysis

To interpret the machine learning models and assess feature contributions, SHapley Additive exPlanations (SHAP) values were calculated. SHAP values provide a consistent measure of feature importance and enable visualization of how individual variables influence model predictions. SHAP analysis focused on the best‐performing model as determined by the C‐index, which was the optimal Cox proportional hazards model in this section (Q4).

### 2.8 Software

All analyses were conducted using R version 4.4.1 (R Foundation for Statistical Computing, Vienna, Austria). ML models were implemented using the mlr3learners and mlr3proba packages. SHAP analysis was performed using the shapviz and kernelshap package.

## 3 Results

### 3.1 Baseline Characteristics of the Participants

A total of 22,308 participants were included in this study. Baseline characteristics stratified by all‐cause and cardiovascular disease (CVD) mortality status are summarized in Table 1. Compared to survivors, individuals who died from all causes or CVD were significantly older (median age: 66.32 vs. 45.17 years for all‐cause mortality; 68.32 vs. 45.17 years for CVD mortality), more likely to be male (52.88% vs. 47.12% for all‐cause; 54.83% vs. 45.17% for CVD), and predominantly of White race.

**TABLE 1:** Baseline characteristics of the participants stratified by all‐cause and CVD mortality.

	All‐cause mortality				CVD mortality			
	Total	Assumed alive	Assumed deceased	*p*	Total	Assumed alive	Assumed deceased	*p*
Age	47.58 ± 0.22	45.17 ± 0.21	66.32 ± 0.40	< 0.0001	46.03 ± 0.21	45.17 ± 0.21	68.83 ± 0.59	< 0.0001
BMI (kg/m^2^)	28.74 ± 0.08	28.77 ± 0.08	28.54 ± 0.15	0.17	28.78 ± 0.08	28.77 ± 0.08	29.17 ± 0.28	0.17
Waist circumference (cm)	98.40 ± 0.20	98.04 ± 0.22	101.36 ± 0.39	< 0.0001	98.21 ± 0.21	98.04 ± 0.22	102.94 ± 0.67	< 0.0001
WHtR	0.58 ± 0.00	0.58 ± 0.00	0.61 ± 0.00	< 0.0001	0.58 ± 0.00	0.58 ± 0.00	0.62 ± 0.00	< 0.0001
Poverty	3.01 ± 0.03	3.06 ± 0.03	2.56 ± 0.05	< 0.0001	3.05 ± 0.03	3.06 ± 0.03	2.55 ± 0.06	< 0.0001
HbA1c	5.59 ± 0.01	5.54 ± 0.01	5.93 ± 0.03	< 0.0001	5.56 ± 0.01	5.54 ± 0.01	6.08 ± 0.05	< 0.0001
Bpxsar	121.74 ± 0.19	120.30 ± 0.19	133.05 ± 0.49	< 0.0001	120.88 ± 0.20	120.30 ± 0.19	136.35 ± 0.87	< 0.0001
Bpxdar	70.64 ± 0.16	70.99 ± 0.17	67.86 ± 0.32	< 0.0001	70.86 ± 0.17	70.99 ± 0.17	67.19 ± 0.64	< 0.0001
WBC (1000cells/μL)	6.79 ± 0.03	6.74 ± 0.03	7.22 ± 0.07	< 0.0001	6.75 ± 0.03	6.74 ± 0.03	7.08 ± 0.07	< 0.0001
Hemoglobin (g/dL)	14.41 ± 0.02	14.44 ± 0.02	14.20 ± 0.05	< 0.0001	14.43 ± 0.02	14.44 ± 0.02	14.12 ± 0.06	< 0.0001
Fast glucose (mmol/L)	5.85 ± 0.02	5.77 ± 0.02	6.50 ± 0.06	< 0.0001	5.81 ± 0.02	5.77 ± 0.02	6.69 ± 0.09	< 0.0001
Fast insulin (pmol/L)	76.94 ± 0.83	75.36 ± 0.86	89.21 ± 2.71	< 0.0001	75.99 ± 0.85	75.36 ± 0.86	92.67 ± 4.17	< 0.0001
Fast triglyceride (mmol/L)	1.49 ± 0.01	1.46 ± 0.01	1.73 ± 0.02	< 0.0001	1.47 ± 0.01	1.46 ± 0.01	1.72 ± 0.04	< 0.0001
HDL cholesterol (mmol/L)	1.39 ± 0.00	1.39 ± 0.01	1.38 ± 0.01	0.85	1.39 ± 0.00	1.39 ± 0.01	1.38 ± 0.01	0.49
LDL cholesterol (mmol/L)	3.00 ± 0.01	3.00 ± 0.01	2.96 ± 0.02	0.06	3.00 ± 0.01	3.00 ± 0.01	2.92 ± 0.04	0.03
C‐reactive protein (mg/dL)	0.41 ± 0.01	0.37 ± 0.01	0.61 ± 0.03	< 0.0001	0.39 ± 0.01	0.37 ± 0.01	0.63 ± 0.06	< 0.0001
Albumin (g/L)	42.54 ± 0.05	42.67 ± 0.05	41.57 ± 0.09	< 0.0001	42.62 ± 0.05	42.67 ± 0.05	41.41 ± 0.14	< 0.0001
METS‐IR	42.89 ± 0.15	42.80 ± 0.16	43.56 ± 0.27	0.01	42.87 ± 0.16	42.80 ± 0.16	44.66 ± 0.49	< 0.001
PA total MET	3408.38 ± 70.07	3558.86 ± 75.79	1815.87 ± 112.41	< 0.0001	3506.61 ± 73.30	3558.86 ± 75.79	1524.45 ± 112.10	< 0.0001
Mcauley	7.57 ± 0.03	7.64 ± 0.03	6.99 ± 0.05	< 0.0001	7.62 ± 0.03	7.64 ± 0.03	6.92 ± 0.09	< 0.0001
Sex								
Female	11,225 (50.69)	9703 (51.14)	1522 (47.12)	< 0.001	10,163 (50.93)	9703 (51.14)	460 (45.17)	< 0.001
Male	11,083 (49.31)	9121 (48.86)	1962 (52.88)		9754 (49.07)	9121 (48.86)	633 (54.83)	
Ethnicity								
Black	4437 (10.48)	3813 (10.56)	624 (9.85)	< 0.0001	4024 (10.58)	3813 (10.56)	211 (11.14)	< 0.0001
Mexican	3931 (8.14)	3473 (8.72)	458 (3.64)		3610 (8.54)	3473 (8.72)	137 (3.77)	
Other	3941 (12.33)	3681 (13.00)	260 (7.11)		3758 (12.79)	3681 (13.00)	77 (7.16)	
White	9999 (69.05)	7857 (67.71)	2142 (79.41)		8525 (68.09)	7857 (67.71)	668 (77.93)	
Marital status								
Married or living with a partner	13,469 (64.04)	11,630 (65.69)	1839 (57.12)	< 0.0001	12,192 (64.63)	11,630 (65.69)	562 (54.34)	< 0.0001
Not married nor living with a partner	8642 (34.91)	7054 (34.31)	1588 (42.88)		7569 (34.38)	7054 (34.31)	515 (45.66)	
Education								
Below high school	6079 (17.61)	4724 (16.10)	1355 (29.50)	< 0.0001	5147 (16.58)	4724 (16.10)	423 (29.77)	< 0.0001
High school and above	16,203 (82.30)	14,083 (83.90)	2120 (70.50)		14,751 (83.33)	14,083 (83.90)	668 (70.23)	
Smoke								
Former	5676 (25.73)	4317 (24.23)	1359 (37.52)	< 0.0001	4739 (24.69)	4317 (24.23)	422 (37.31)	< 0.0001
Never	11,927 (52.75)	10,532 (54.60)	1395 (38.63)		11,020 (54.21)	10,532 (54.60)	488 (44.67)	
Now	4685 (21.46)	3959 (21.17)	726 (23.85)		4141 (21.04)	3959 (21.17)	182 (18.02)	
Alcohol use								
Former	3594 (13.60)	2492 (12.58)	1102 (31.69)	< 0.0001	2849 (12.27)	2492 (12.58)	357 (33.34)	< 0.0001
Never	2811 (10.15)	2291 (10.56)	520 (14.78)		2474 (9.90)	2291 (10.56)	183 (16.68)	
Now	13,750 (68.13)	12,134 (76.86)	1616 (53.53)		12,624 (69.62)	12,134 (76.86)	490 (49.98)	
Hypertension								
No	12,742 (62.43)	11,709 (66.24)	1033 (32.94)	< 0.0001	11,957 (64.72)	11,709 (66.24)	248 (25.22)	< 0.0001
Yes	9561 (37.55)	7112 (33.76)	2449 (67.06)		7956 (35.25)	7112 (33.76)	844 (74.78)	
DM								
No	17,943 (85.43)	15,658 (87.53)	2285 (69.09)	< 0.0001	16,337 (86.67)	15,658 (87.53)	679 (63.92)	< 0.0001
Yes	4365 (14.57)	3166 (12.47)	1199 (30.91)		3580 (13.33)	3166 (12.47)	414 (36.08)	
Hyperlipidemia								
No	5948 (27.76)	5317 (29.12)	631 (17.20)	< 0.0001	5492 (28.63)	5317 (29.12)	175 (15.63)	< 0.0001
Yes	16,360 (72.24)	13,507 (70.88)	2853 (82.80)		14,425 (71.37)	13,507 (70.88)	918 (84.37)	
ASCVD								
No	19,992 (91.75)	17,495 (94.16)	2497 (72.98)	< 0.0001	18,184 (93.04)	17,495 (94.16)	689 (63.39)	< 0.0001
Yes	2314 (8.25)	1327 (5.84)	987 (27.02)		1731 (6.96)	1327 (5.84)	404 (36.61)	

Abbreviations: ASCVD, atherosclerotic cardiovascular disease; BMI, body mass index; BPXDAR, DBP average reported to examinee; BPXSAR, SBP average reported to examinee; DM, diabetes mellitus; HDL, high‐density lipoprotein; LDL, low‐density lipoprotein; MET, metabolic equivalent; METS‐IR, Metabolic Score for Insulin Resistance; PA, physical activity; WBC, white blood cell; WHtR, waist‐to‐height ratio.

Deceased participants also exhibited a higher prevalence of smoking (38.63% never smokers vs. 61.37% ever smokers for all‐cause; 44.67% vs. 55.33% for CVD) and lower levels of physical activity (median MET‐min/week: 1815.87 vs. 3558.86 for all‐cause; 1524.45 vs. 3558.86 for CVD). Among CVD‐related deaths, hyperlipidemia was the most common comorbidity, present in 82.80% of all‐cause deaths and 84.37% of CVD deaths.

Metabolic differences were also apparent: deceased individuals had greater central adiposity, reflected by higher waist circumference (101.36 vs. 98.04 cm) and waist‐to‐height ratio (WHtR, 0.61 vs. 0.58), as well as elevated HbA1c levels (5.93% vs. 5.54%) and C‐reactive protein (0.61 vs. 0.37 mg/dL), despite similar BMI values (28–29 kg/m^2^). Insulin resistance indices also varied by outcome: the McAuley index was significantly lower in deceased individuals (6.99 vs. 7.64; *p* < 0.0001), while METS‐IR was significantly higher in those with CVD mortality (43.56 vs. 42.80; *p* = 0.01).

Socioeconomic disparities were evident. Deceased individuals more frequently had an education level below high school (29.50% vs. 16.10%) and were more likely to live without a partner (42.88% vs. 34.31%). Patterns of alcohol use also differed, with higher rates of former or heavy drinking observed in mortality groups.

### 3.2 Predictive Value of McAuley Index and METS‐IR for All‐Cause and Cardiovascular Mortality

**FIGURE 2:**
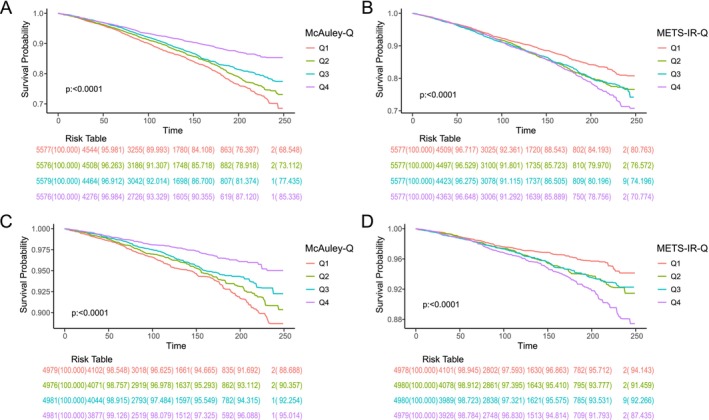
Kaplan–Meier (KM) survival curves for all‐cause (A, B) and cardiovascular (C, D) mortality stratified by the McAuley index (A, C) and METS‐IR (B, D). METS‐IR, metabolic syndrome insulin resistance index.

During a median follow‐up of 110 ± 66.0 months, 3484 all‐cause deaths and 1093 cardiovascular deaths were recorded among the 22,308 participants. The all‐cause and cardiovascular mortality rates were 16.3 and 5.1 per 1000 person‐years, respectively. Kaplan–Meier survival curves revealed significant differences in all‐cause mortality across quartiles of both the McAuley index and METS‐IR. Specifically, the lowest quartile (Q1) of the McAuley index and the highest quartile (Q4) of METS‐IR were associated with the highest all‐cause mortality risks (log‐rank *p* < 0.001 and *p* = 0.008, respectively; Figure 2A,B). Similar patterns were observed for cardiovascular mortality, with the worst survival outcomes again seen in Q1 of the McAuley index and Q4 of METS‐IR (log‐rank *p* < 0.001 for both; Figure 2C,D).

Associations between the McAuley index and METS‐IR and all‐cause mortality were further examined using Cox proportional hazards regression (Table S2). As a continuous variable, the McAuley index was significantly inversely associated with all‐cause mortality in both unadjusted and partially adjusted models. In the crude model, each 1‐unit increase in the McAuley index corresponded to a 9.2% reduction in all‐cause mortality risk (hazard ratio [HR]: 0.91, 95% confidence interval [CI]: 0.89–0.93; *p* < 0.0001). In the fully adjusted model (Model 3), although the inverse trend persisted, statistical significance was not retained. When analyzed by quartiles, Q2–Q4 showed a significantly lower risk compared to Q1 in unadjusted models, but the associations were attenuated after adjusting for potential confounders, including hypertension, hyperlipidemia, and diabetes.

In contrast, METS‐IR as a continuous variable remained significantly associated with increased all‐cause mortality in all models, with HRs ranging from 1.01 – 1.03 (*p* < 0.05). The highest quartile (Q4) of METS‐IR was also significantly associated with increased all‐cause mortality in the unadjusted model (HR: 1.32, 95% CI: 1.17–1.49; *p* < 0.0001), though this association lost significance after covariate adjustment.

Cox models also demonstrated significant associations between both indices and cardiovascular mortality (Table S3). Notably, the McAuley index showed the strongest association, with each 1‐unit increase corresponding to an 11.3% reduction in cardiovascular mortality risk (*p* < 0.001).

### 3.3 Nonlinear U‐Shaped Associations Between Insulin Resistance Indices and Mortality

**FIGURE 3:**
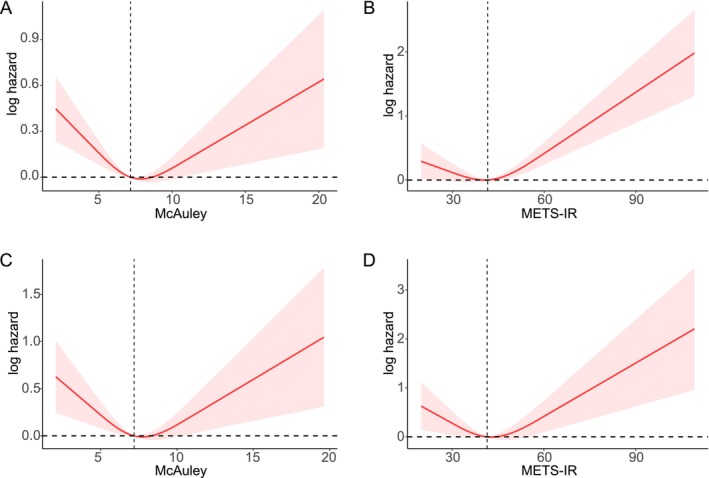
Associations between the McAuley index and METS‐IR with all‐cause and cardiovascular mortality. Restricted cubic spline (RCS) plots show the relationship of the McAuley index (A, C) and METS‐IR (B, D) with all‐cause (A, B) and cardiovascular (C, D) mortality. METS‐IR, metabolic syndrome insulin resistance index.

Restricted cubic spline (RCS) analysis based on multivariable Cox regression was performed to explore potential nonlinear associations between the McAuley index, METS‐IR, and mortality outcomes. After adjusting for covariates, significant U‐shaped relationships were observed between both indices and all‐cause (nonlinear *p* < 0.001) and cardiovascular mortality (nonlinear *p* < 0.05), as shown in Figure 3.

### 3.4 Consistent Prognostic Performance of Insulin Resistance Indices Across Clinical Subgroups

**FIGURE 4:**
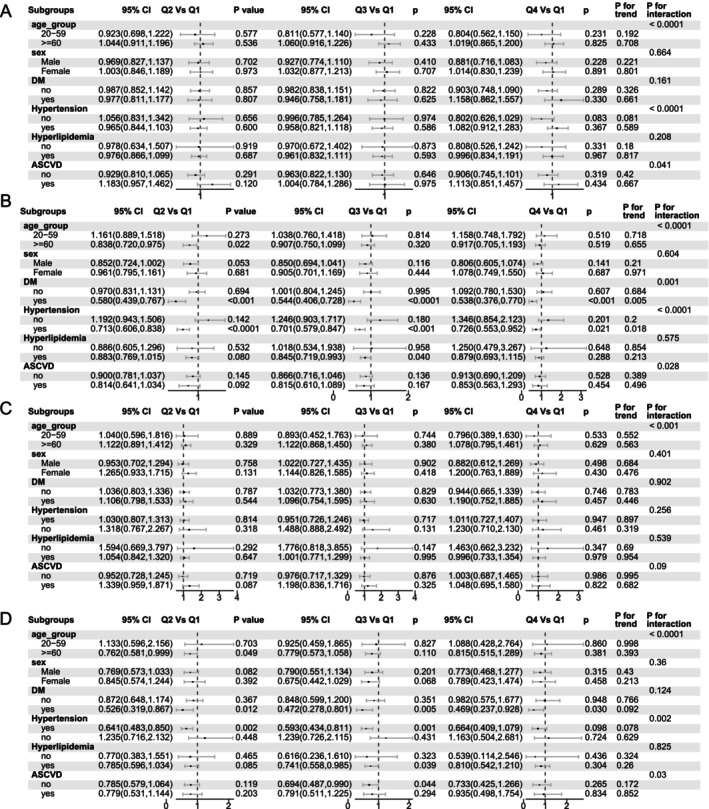
Subgroup analysis of the associations between the McAuley index and METS‐IR with all‐cause and cardiovascular mortality. Forest plots show hazard ratios for the McAuley index (A, C) and METS‐IR (B, D) with respect to all‐cause (A, B) and cardiovascular (C, D) mortality. Models were adjusted for age, sex, diabetes, hypertension, hyperlipidemia, and atherosclerotic cardiovascular disease.

Subgroup analysis stratified by age, sex, diabetes status, hypertension, hyperlipidemia, and atherosclerotic cardiovascular disease (ASCVD) demonstrated that the prognostic associations of the McAuley index and METS‐IR with all‐cause and cardiovascular mortality were largely consistent across subgroups (Figure 4A–D). However, certain interactions were observed, suggesting that the prognostic impact of both indices was more pronounced among older adults and individuals with hypertension (Figure 4A,B).

### 3.5 Machine Learning Models Integrating Insulin Resistance Indices for Mortality Risk Prediction

**FIGURE 5:**
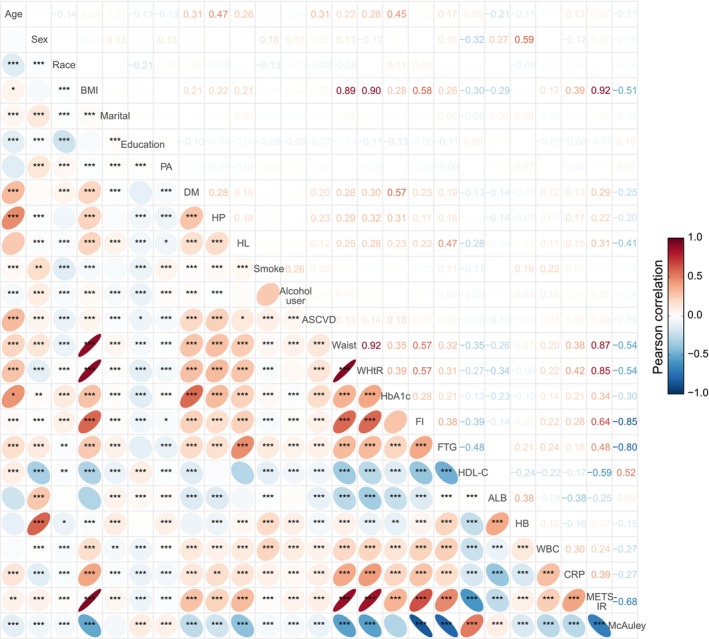
Pearson correlation heatmap of baseline clinical variables. Correlation coefficients are shown above each cell. ALB, albumin; AU, alcohol use; CRP, C‐reactive protein; DM, diabetes mellitus; FI, fasting insulin; FTG, fasting triglycerides; HDL‐C, high‐density lipoprotein cholesterol; HL, hyperlipidemia; HP, hypertension; PA, physical activity. Significance levels: *p < 0.05; **p < 0.01; ***p < 0.001.

To enhance risk stratification, we applied feature selection based on correlation analysis. In addition to adiposity‐related indices (waist circumference, WHtR, and BMI), METS‐IR was positively correlated with fasting insulin and triglycerides (Figure 5). Conversely, the McAuley index showed an inverse pattern compared to METS‐IR. Both indices demonstrated moderate correlations with baseline characteristics, including age, sex, education, physical activity, smoking status, and alcohol consumption, supporting their inclusion as independent variables alongside these features (Figure 5).

**FIGURE 6:**
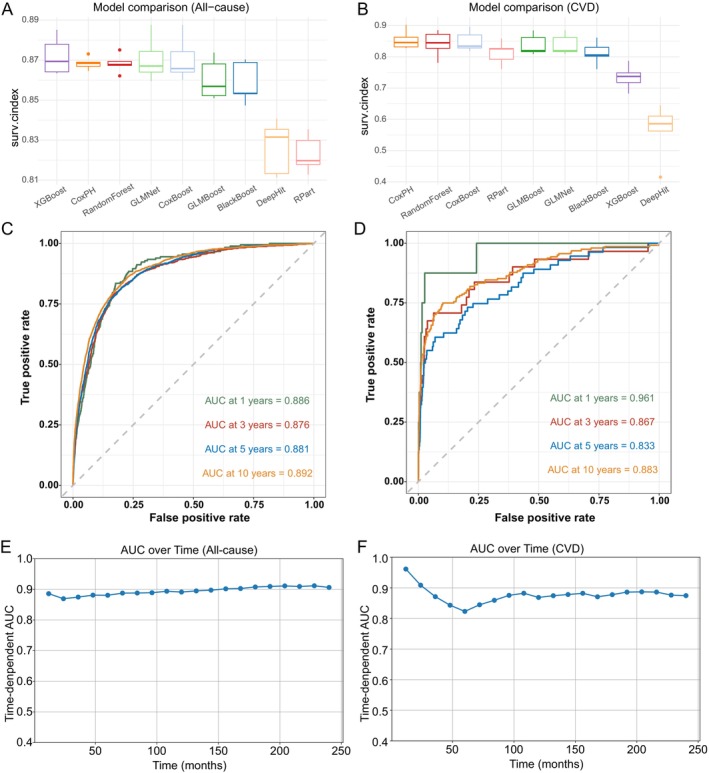
Performance of machine learning models in predicting all‐cause and cardiovascular mortality. (A, B) Boxplots of C‐index values across 5‐fold cross‐validation for nine machine learning algorithms in all‐cause (A) and cardiovascular (B) mortality. (C, D) Time‐dependent ROC curves at 1, 3, 5, and 10 years for the best‐performing models in all‐cause (C) and cardiovascular (D) mortality. (E, F) Time‐dependent AUC curves are shown for (E) all‐cause mortality and (F) CVD mortality, evaluated from 1 to 20 years of follow‐up.

We constructed predictive models using nine machine learning algorithms with repeated five‐fold cross‐validation. As shown in Table S1, for all‐cause mortality, the optimized XGBoost model achieved the highest performance with a C‐index of 0.872 (95% CI: 0.860–0.884). CoxPH (C‐index: 0.868, 95% CI: 0.864–0.872) and other tuned models, including GLMNet (0.871) and CoxBoost (0.870), demonstrated highly comparable performance. For cardiovascular mortality, CoxPH remained the top‐performing model, yielding a C‐index of 0.854 (95% CI: 0.816–0.892) (Figure 6A,B). Given its robustness, interpretability, and marginal differences from optimized ML models (< 0.004), CoxPH was selected as the primary model. The time‐dependent AUCs were 0.887, 0.878, 0.882, and 0.893 at 1, 3, 5, and 10 years for all‐cause mortality, and 0.916, 0.867, 0.833, and 0.883, respectively, for cardiovascular mortality (Figure 6C,D). Extended analysis from 1 to 20 years revealed that the time‐dependent AUC for all‐cause mortality was remarkably stable (range: 0.87–0.91), with its predictive performance strengthening over the long term. The AUC consistently exceeded 0.9 after 13 years, peaking at 19 years (Figure 6E). For cardiovascular mortality, the CoxPH model also demonstrated superior predictive accuracy (C‐index: 0.854, 95% CI: 0.816–0.892). While a more granular temporal analysis up to 20 years showed that the time‐dependent AUC exhibited greater variation (range: 0.82–0.96) and a peak at 1 year, its long‐term predictive ability remained excellent and stable, maintaining an AUC of approximately 0.88 after 13 years throughout the remainder of the follow‐up period (Figure 6F).

### 3.6 SHAP Analysis Reveals Key Predictors of Mortality in Machine Learning Models

**FIGURE 7:**
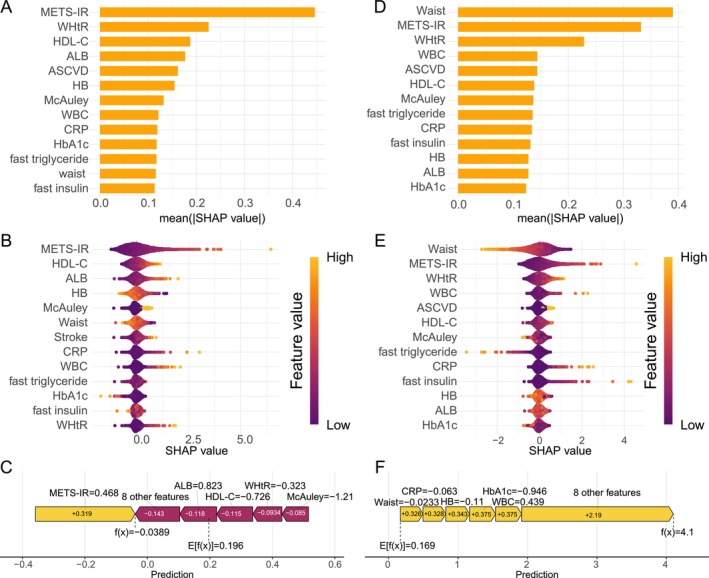
SHAP analysis for Cox proportional hazards models predicting all‐cause and cardiovascular mortality. (A) Mean absolute SHAP values indicating feature importance for all‐cause mortality. (B) SHAP value distributions across predictor ranges for all‐cause mortality (color indicates feature value: purple = low, yellow = high). (C) Force plot illustrating feature contributions to an individual prediction of all‐cause mortality (*f*(*x*) = −0.0389; baseline E[*f*(*x*)] = 0.196). (D) Mean absolute SHAP values for predictors of cardiovascular mortality. (E) SHAP value distributions for cardiovascular mortality predictors. (F) Force plot illustrating feature contributions to an individual prediction of cardiovascular mortality (*f*(*x*) = 4.1; baseline E[*f*(*x*)] = 0.169). ALB, albumin; ASCVD, atherosclerotic cardiovascular disease; CRP, C‐reactive protein; HB, hemoglobin; HbA1c, glycated hemoglobin; HDL‐C, high‐density lipoprotein cholesterol; METS‐IR, metabolic syndrome insulin resistance index; WBC, white blood cell count; WHtR, waist‐to‐height ratio.

Given the strong predictive performance of the robust and parsimonious CoxPH model, we applied SHAP analysis to provide a complementary layer of interpretability. Unlike hazard ratios, which offer a single global value, SHAP provided an intuitive visualization of each predictor's relative contribution and direction. This method also served as a valuable diagnostic tool, confirming the linearity assumption for our key insulin resistance indices (McAuley Index and METS‐IR), ultimately reinforcing the robustness of our results. For all‐cause mortality, METS‐IR emerged as the most influential predictor (mean|SHAP value| = 0.45), followed by WHtR (0.23), HDL‐C (0.19), and albumin (ALB; 0.18) (Figure 7A). Directionality analysis revealed that higher METS‐IR values were consistently associated with increased mortality risk, whereas elevated HDL‐C levels exhibited a protective effect (Figure 7B). Other key predictors included ALB, atherosclerotic cardiovascular disease (ASCVD) risk score, and hemoglobin (HB), each exerting moderate influence. An illustrative individual prediction force plot demonstrated that METS‐IR (SHAP value = 0.468) and ALB (0.823) increased risk, while HDL‐C (−0.726) and the McAuley index (−1.21) had protective effects, culminating in a net prediction of −0.0389 from a baseline of 0.196 (Figure 7C).

For cardiovascular mortality, waist circumference was the most important predictor (mean|SHAP value| = 0.39), followed closely by METS‐IR (0.33) and WHtR (0.23) (Figure 7D). SHAP distribution plots indicated that increased waist circumference and METS‐IR were associated with higher CVD mortality risk, while low ALB and HB levels also conferred elevated risk (Figure 7E). Inflammatory biomarkers such as CRP and WBC contributed moderately to prediction accuracy. The force plot for an individual prediction illustrated how anthropometric and metabolic variables collectively drove a high‐risk estimate (*f*(*x*) = 4.1), emphasizing their role in cardiovascular risk stratification (Figure 7F).

These findings underscore the distinct yet overlapping predictive importance of metabolic, anthropometric, and inflammatory markers in differentiating all‐cause from cardiovascular‐specific mortality.

## 4 Discussion

In this comprehensive analysis of two decades of NHANES data (1999–2018), we demonstrated that both the McAuley index and METS‐IR were significant and independent predictors of all‐cause and cardiovascular mortality among individuals with established cardiovascular disease (CVD). Specifically, a lower McAuley index (indicating greater insulin resistance) and higher METS‐IR scores were consistently associated with an increased risk of mortality. The restricted cubic spline (RCS) analysis further revealed significant U‐shaped associations, highlighting non‐linear risk patterns. By leveraging nine machine learning algorithms, we developed robust prediction models, with the Cox proportional hazards (Coxph) model achieving the highest performance. SHAP (SHapley Additive exPlanations) analysis offered interpretable insights, identifying METS‐IR as a key contributor to mortality risk and waist circumference as the most influential predictor for cardiovascular‐specific mortality.

Our findings extend prior research linking insulin resistance to adverse cardiovascular outcomes (Montaigne et al. 2021; Rohm et al. 2022). The McAuley index, which incorporates fasting insulin and triglyceride levels, has previously been associated with cardiovascular risk in general populations (Hochsmann et al. 2021; Petersen et al. 2024). Our study adds novel evidence by validating its prognostic relevance in patients with pre‐existing CVD, suggesting that insulin resistance remains an important mortality risk factor even in late‐stage disease. Notably, each unit increase in the McAuley index was associated with an 11.3% reduction in cardiovascular mortality, underscoring its clinical utility. Similarly, METS‐IR—a novel surrogate marker that does not rely on insulin assays—demonstrated comparable predictive power. Given its ease of calculation and independence from insulin measurement, METS‐IR offers a practical and scalable alternative for use in resource‐limited settings or routine clinical practice (Liu et al. 2025).

The non‐linear relationships between these indices and mortality risk, as revealed by RCS plots, reflect the complex, threshold‐dependent effects of insulin resistance on long‐term outcomes. The L‐shaped curves suggest disproportionately higher risks at the lower end of the McAuley index and the higher end of METS‐IR, implying potential thresholds for clinical intervention. These findings support the use of flexible modeling techniques—such as machine learning—to better capture the intricate biology underlying metabolic dysfunction and mortality.

Our machine learning models, particularly the Coxph algorithm, achieved C‐indices of 0.868 and 0.854 for all‐cause and cardiovascular mortality, respectively. These results are consistent with the growing body of evidence that machine learning techniques outperform traditional statistical models in capturing non‐linear relationships and high‐order interactions in biomedical data (Leuch et al. 2016; Liu et al. 2022; Manduchi et al. 2022). Despite concerns regarding the interpretability of machine learning (“black box” issue), our application of SHAP analysis provided clinically meaningful explanations for model predictions (Wang et al. 2025). The application of SHAP analysis to the Cox model provided a model‐agnostic view of feature importance, which aligned perfectly with the hazard ratios and further confirmed the primary role of McAuley Index and METS‐IR as the dominant predictor.

SHAP analysis highlighted that while conventional cardiovascular risk factors (e.g., hypertension, age, diabetes) remained important, metabolic and anthropometric indicators contributed substantial additional predictive value. For all‐cause mortality, METS‐IR was the most influential predictor, followed by waist‐to‐height ratio (WHtR), HDL‐C, and albumin. In contrast, for cardiovascular mortality, waist circumference emerged as the most powerful predictor, followed by METS‐IR and WHtR. These results emphasize the prominent role of central adiposity and metabolic dysfunction in determining long‐term outcomes in individuals with CVD. Notably, our findings reinforce the growing recognition that measures of central obesity (e.g., waist circumference, WHtR) may surpass traditional metrics like BMI in cardiovascular risk assessment (Powell‐Wiley et al. 2021; Rubino et al. 2025).

Our analysis revealed that the CoxPH model outperformed several advanced ML algorithms in predicting all‐cause and cardiovascular mortality. This finding indicates that the relationships between insulin resistance indices (McAuley Index and METS‐IR) and mortality are predominantly linear and additive, which the Cox model captures with high statistical efficiency. Given that these indices are derived from well‐established physiological variables with strong, largely linear associations with cardiovascular risk, additional model complexity provided limited benefit and even risked overfitting despite a large cohort size (Christodoulou et al. 2019). This result aligns with the “No Free Lunch” theorem (Yang et al. 2025) and prior benchmarking studies showing that in tabular medical data, traditional or semi‐parametric models often match or exceed more complex ML approaches (Tennenhouse et al. 2020; van der Ploeg et al. 2014). Importantly, the application of ML methods in our study was not redundant: tree‐based models achieved high accuracy (C‐index > 0.80) and SHAP analysis consistently identified insulin resistance indices as the top predictors. This convergence across CoxPH and ML models strengthens the robustness of our conclusions. In summary, the superior performance of CoxPH reflects the clarity of the biological signal, not a limitation of ML. For risk stratification based on established biomarkers, simpler and more interpretable models may be optimal, while ML approaches may offer greater advantages in settings with more complex or non‐linear data types.

The observed differences in predictor importance between all‐cause and cardiovascular mortality may reflect distinct pathophysiological pathways. While systemic metabolic dysfunction—captured by METS‐IR—may be the primary driver of general mortality, visceral adiposity appears more directly implicated in cardiovascular‐specific outcomes. This distinction has important clinical implications: it supports tailored risk stratification strategies and highlights central obesity as a modifiable therapeutic target in patients with established CVD.

From a clinical perspective, our findings support the integration of insulin resistance indices such as METS‐IR and the McAuley index into routine risk assessment for patients with cardiovascular disease. These indices are derived from widely available biomarkers, making them cost‐effective and easily implementable tools. Furthermore, their prognostic utility suggests potential for early intervention in high‐risk patients—especially in primary care or community health settings—where advanced diagnostic tools may be limited.

The strengths of our study include the use of a nationally representative sample with long‐term follow‐up, adjustment for a wide range of confounding factors, comprehensive modeling strategies, and the use of interpretable machine learning techniques. The large sample size (*n* = 22,308) of patients with established CVD provides robust power for detecting clinically relevant associations. Moreover, our parallel use of both parametric and non‐parametric models enabled consistent and validated insights.

Nevertheless, several limitations should be acknowledged. First, the observational design of NHANES precludes causal inference. Second, insulin resistance indices were only measured at baseline, and dynamic changes over time were not accounted for. Third, although we adjusted for numerous covariates, residual confounding may persist. Fourth, self‐reported medical history in NHANES could introduce recall bias. Lastly, the generalizability of our machine learning models to non‐U.S. populations requires further validation.

Future research should aim to externally validate these findings in prospective cohorts across diverse populations. Investigations into interventions targeting insulin resistance and central adiposity are warranted to determine whether modifying these risk factors can improve clinical outcomes. Finally, efforts should be made to translate these indices into simple, point‐of‐care tools for routine clinical use.

## 5 Conclusions

In conclusion, this study highlights the significant prognostic value of insulin resistance indices—particularly the McAuley index and METS‐IR—for predicting all‐cause and cardiovascular mortality. Our findings demonstrate that these indices exhibit strong, non‐linear associations with long‐term outcomes and can be effectively integrated into predictive models using machine learning approaches. METS‐IR, in particular, emerged as a robust and accessible biomarker, offering a practical tool for mortality risk stratification in diverse clinical settings. The interpretability of our models via SHAP analysis enhances their translational potential, providing clear insights into individual‐level risk contributors. These results support the routine assessment of metabolic dysfunction and central adiposity in patients with cardiovascular disease and underscore the need for targeted interventions aimed at mitigating insulin resistance to improve clinical outcomes.

## Author Contributions

**Zeyi Zhou:** conceptualized the study, performed data curation, and wrote the original draft. **QiuJu Ding:** conducted formal analysis, designed the methodology, and performed the visualization of results. **Xinlong Tang:** responsible for software implementation, validation, and data interpretation. **Lixiang Han:** contributed to data collection and preprocessing. **Yali Wang:** performed literature review and assisted with statistical analysis. **Jintao Qian:** contributed to project administration and resources. **Kai Li:** provided funding acquisition and project administration. **Qing Zhou:** responsible for study design and critical revision of the manuscript.

## Ethics Statement

This study was conducted in accordance with the principles of academic integrity and ethical research. All data was obtained through publicly available sources or with proper authorization, and no personal, confidential, or sensitive information was collected or used without informed consent. The authors affirm that the research process complied with institutional and international ethical standards, and that there were no conflicts of interest that could have influenced the outcomes of this work. No experiments involving human participants or animals were conducted in this study.

## Consent

All participants have agreed that their data would be included in the NHANES database and could be provided to researchers around the world for academic research.

## Conflicts of Interest

The authors declare no conflicts of interest.

## Supporting information


**Table S1:** fsn371080‐sup‐0001‐TableS1.xlsx.

**Table S2:** fsn371080‐sup‐0002‐TableS2.xlsx.

**Table S3:** fsn371080‐sup‐0003‐TableS3.xlsx.

## Data Availability

The data that support the findings of this study are openly available in NIH at https://www.cdc.gov/nchs/nhanes/index.html.
